# Concise Review: Considering Optimal Temperature for Short-Term Storage of Epithelial Cells

**DOI:** 10.3389/fmed.2021.686774

**Published:** 2021-08-18

**Authors:** Ayyad Zartasht Khan, Tor Paaske Utheim, Catherine Joan Jackson, Kim Alexander Tønseth, Jon Roger Eidet

**Affiliations:** ^1^Department of Medical Biochemistry, Oslo University Hospital, Oslo, Norway; ^2^Department of Surgery, Sørlandet Hospital Arendal, Arendal, Norway; ^3^Department of Ophthalmology, Sørlandet Hospital Arendal, Arendal, Norway; ^4^Institute of Clinical Medicine, Faculty of Medicine, University of Oslo, Oslo, Norway; ^5^Department of Oral Biology, Faculty of Dentistry, University of Oslo, Oslo, Norway; ^6^Department of Ophthalmology, Stavanger University Hospital, Stavanger, Norway; ^7^Department of Plastic and Reconstructive Surgery, Oslo University Hospital, Oslo, Norway; ^8^Department of Ophthalmology, Oslo University Hospital, Oslo, Norway; ^9^Ifocus Eye Clinic, Haugesund, Norway

**Keywords:** cell banking, regenerative medicine, storage temperature, cell therapy, storage technologies, transplantation

## Abstract

Transplantation of novel tissue-engineered products using cultured epithelial cells is gaining significant interest. While such treatments can readily be provided at centralized medical centers, delivery to patients at geographically remote locations requires the establishment of suitable storage protocols. One important aspect of storage technology is temperature. This paper reviews storage temperature for above-freezing point storage of human epithelial cells for regenerative medicine purposes. The literature search uncovered publications on epidermal cells, retinal pigment epithelial cells, conjunctival epithelial cells, corneal/limbal epithelial cells, oral keratinocytes, and seminiferous epithelial cells. The following general patterns were noted: (1) Several studies across different cell types inclined toward 4 and 16°C being suitable short-term storage temperatures. Correspondingly, almost all studies investigating 37°C concluded that this storage temperature was suboptimal. (2) Cell death typically escalates rapidly following 7–10 days of storage. (3) The importance of the type of storage medium and its composition was highlighted by some of the studies; however, the relative importance of storage medium vs. storage temperature has not been investigated systematically. Although a direct comparison between the included investigations is not reasonable due to differences in cell types, storage media, and storage duration, this review provides an overview, summarizing the work carried out on each cell type during the past two decades.

## Introduction

**Figure 1:**
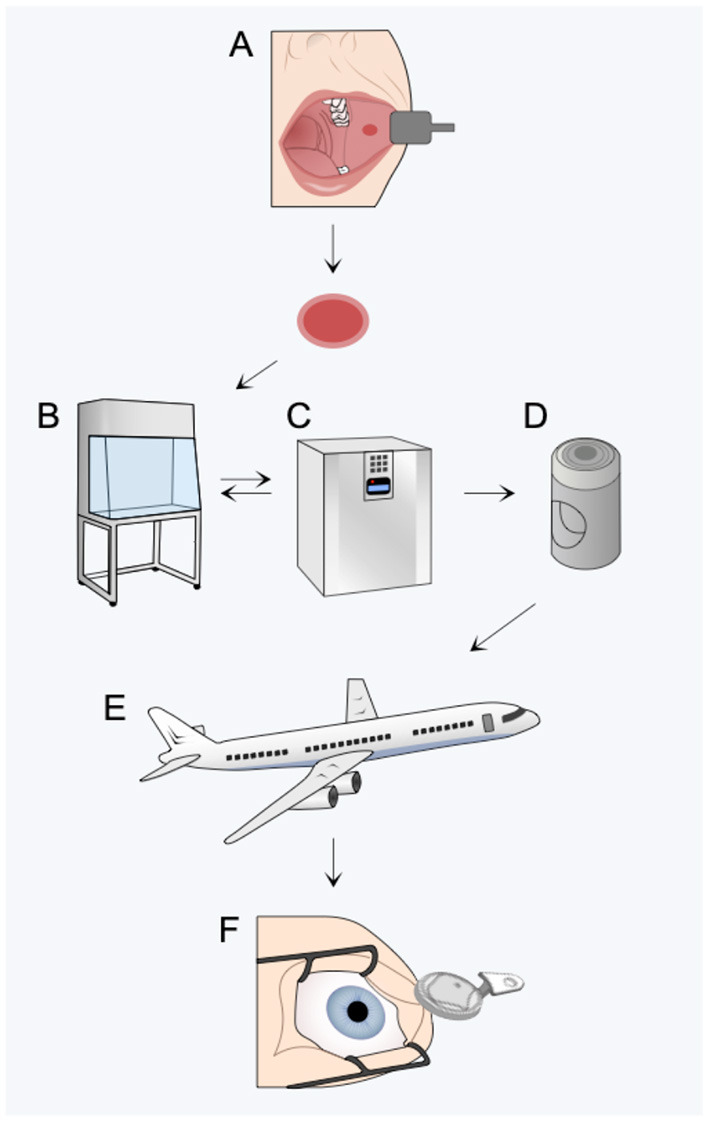
Optimizing storage technologies can improve access to novel regenerative medicine therapies by ensuring graft quality throughout harvest **(A)**, manipulation **(B)**, expansion **(C)**, packaging **(D)**, transport **(E)**, and transplantation **(F)**.

Transplantation of skin grafts is common in clinical practice. However, novel tissue-engineered therapies using cultured epithelial cells are gaining significant interest due to several breakthroughs during the past few decades. Applications include regeneration of burn wounds (1), corneal diseases (2), urethral reconstruction (3), and treatment of retinal dysfunction (4). Provision of these therapies at centralized medical centers has been accomplished, but delivery of such treatment opportunities to patients at geographically remote locations is equally important from a health equity perspective. Because tissue-engineering laboratories require specialized facilities and are subject to high safety and quality standards, few laboratories are able to meet these requirements. This will likely be a barrier to widespread access to such therapies. Therefore, tissue storage technologies need to be improved in order to facilitate transportation of novel tissue-engineered products from centralized laboratories to clinics worldwide (5) (Figure 1). Optimization of tissue storage technology can also facilitate greater flexibility in surgery logistics and allow sufficient time for quality control and microbiological testing (5).

Temperature is generally considered important in transplantation medicine when transporting (1) donor tissue from the operating room to the laboratory and (2) the manufactured tissue-engineered product from the laboratory back to the operating room. While the latter step has been the subject of many investigations, the former step has received little attention. Due to this lack of evidence, this review will focus on storage temperature in general. To maintain storage temperature during transport, thermal insulation inserts, chilled coolant packs, and refrigerated transportation can be employed. Additionally, sophisticated purpose-built storage devices have been described (6, 7).

There are chiefly three approaches to storage of mammalian cells (8): (1) cryopreservation, (2) cell desiccation, and (3) hypothermic cell preservation. While cryopreservation is useful in laboratory settings, it presents logistical challenges during transport (9) and is associated with low post-thaw cell viability (10) and dimethyl sulfoxide (DMSO)-associated toxicity. Desiccation involves preserving cells in a dehydrated form by either freeze-drying or using a vacuum, but concerns related to exposure of cells to severe osmotic imbalance (11, 12), free radical-mediated cell toxicity (13), and chemical treatment of cells before transplantation (14) are important disadvantages. Hypothermic cell preservation (at above freezing temperature) slows down cell metabolism without causing cellular ice damage, is practical, and is a method already widely in use (15). Whereas the limited storage time (in comparison to cryopreservation) remains a significant drawback, the use of biomaterials, such as hydrogels, has made this storage technique very relevant because it enables the delivery of cultured cells in a structurally inert way without compromising graft pliability or biocompatibility (16–18).

This study reviews the scientific literature reported during the past two decades on epithelial cell storage at above freezing point in an effort to uncover the optimal temperature for hypothermic storage of epithelial cells. Studies on the storage of donor corneas are excluded from the review, as this has been adequately covered elsewhere (19, 20).

## Methods

**Figure 2:**
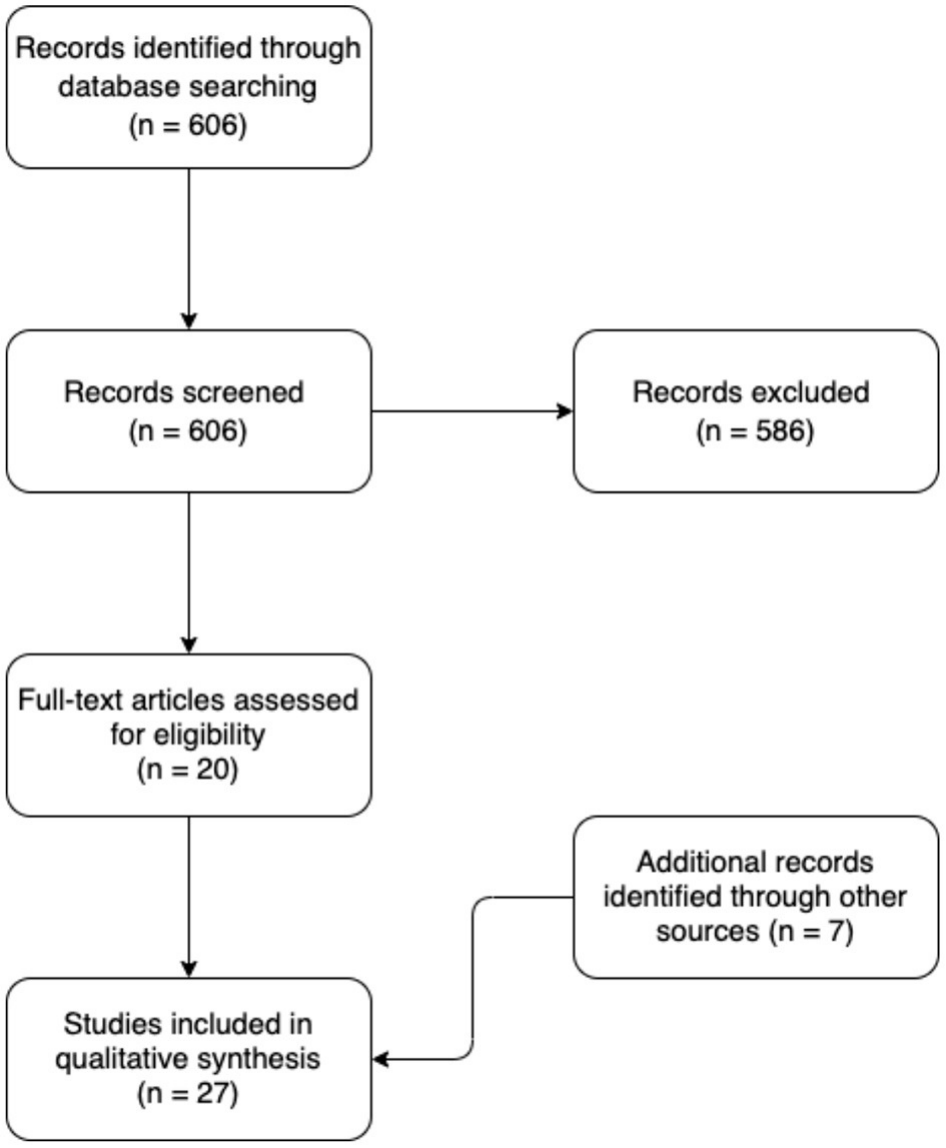
Flow diagram of the literature search.

We performed a literature search on the storage of epithelial cells. With the search algorithms provided in the Appendix, the following databases were searched: Embase, Ovid MEDLINE, Cochrane Library, and Google Scholar. A total of 606 records were retrieved through database searching. These records were then screened manually. Articles were excluded according to predetermined criteria, i.e., if they were conducted on non-human tissue, if they were performed on non-epithelial cells, if they primarily were focused on storage of donor corneas, or if the storage technique was other than above-0°C storage, such as cryopreservation. Also excluded were patents, dissertations, articles in languages other than English, and papers published prior to year 2000. Twenty records remained eligible following the exclusion process. Additionally, seven records were identified based on the authors' acquaintance with the subject matter. These were not detected by the literature search. Thus, 27 English-language publications from the last two decades investigating above-freezing point storage of human epithelial cells were included in the final qualitative synthesis (Figure 2; Table 1).

**Table 1:** Non-freezing storage of epithelial cells.

**References**	**Type of epithelial tissue**	**Transplant type**	**Storage duration**	**Storage medium**	**Storage temperature(s) investigated**	**Viability**	**Morphology**	**Phenotype**
**Epidermal cells**								
Ringstad et al. (21)	Epidermal cells	CCS	15 days	MEM-based	12°C	Viability deteriorates by storage day 11.	Morphology is disrupted during extended storage but improves with reincubation	Reincubated CES stored for 15 days retained proliferative function and the ability to differentiate.
Reppe et al. (22)	Epidermal cells	CCS	Up to 11 days	CnT Prime and MEM-based	12°C	MEM-based storage media showed better viability compared to CnT Prime. Mathematic simulations suggested glycerol and fenoldopam mesylate as viability-promoting storage media additives.	Carnosine, fenoldopam mesylate, and glycerol had a beneficial effect on morphology when used as storage media additives.	NA
Jackson et al. (23)	Epidermal cells	CCS	7 days	MEM-based	4, 8, 12, 16, and 24°C	Optimal storage temperatures for viability: 12 and 16°C	Optimal storage temperature for morphology: 12°C.	12°C storage demonstrated best preservation of undifferentiated cell phenotype.
Jackson et al. (24)	Epidermal cells	CCS	14 days	MEM-based	4, 8, 12, 16, 20, 24, 28, 32, and 37°C	NA	NA	Lower storage temperatures, and in particular 12°C, were optimal in preserving an undifferentiated phenotype during storage.
Jackson et al. (25)	Epidermal cells	CCS	14 days	MEM-based	4, 8, 12, 16, 20, 24, 28, 32, and 37°C	Optimal storage temperature for viability: 24°C. However, almost 60% cell viability was conserved at 12, 28, 32, and 37°C.	Optimal storage temperatures for morphology: 12 and 16°C	Storage at 12 and 20°C preserved proliferative function at a similar level as the non-stored control.
Knapik et al. (26)	Human skin	Split-thickness skin grafts	Up to 7 days	Saline-moisturized gauze	4°C	Viability dropped to 44% after 3 days of storage and remained at this level during the subsequent days.	No changes observed.	NA
Li et al. (27)	Human skin	Split-thickness skin grafts and keratinocytes	Up to 28 days	Saline, Hartmann's solution, DMEM, and DMEM/F12	4°C	Viability decreased proportionally with storage time.	NA	Compared to DMEM-based media, storage in saline and Hartmann's solution resulted in better post-storage keratinocyte proliferative capacity.
Seet et al. (28)		Tissue-engineered skin construct composed of keratinocytes and fibroblasts	3 days	DMEM/F12	4°C	Viability was 95% before storage, 91% after 24 h storage, 92% after 48 h storage, and 91% after 72 h storage.	No change in morphology during storage.	No significant difference was found in gene expression.
Robb et al. (29)	Human skin	Split-thickness skin grafts and skin biopsies	Up to 21 days	Saline and MEM	4°C or room temperature	NA	Skin stored in MEM-based media maintained better histologic anatomy than skin stored in saline.	NA
**Retinal pigment epithelial cells**								
Islam et al. (30)	ARPE-19	CCS	21 days	MEM-based	4, 16, and 37°C	Optimal storage temperature for viability: 16°C	Morphology was best preserved at 16°C.	Dedifferentiation was noted in all storage temperatures.
Kitahata et al. (31)	hiPSC-RPE	Cell suspensions	Up to 120 h	DMEM-based	4, 16, 25, and 37°C	Following 120 h of storage, highest viability was achieved at 16°C storage	Cells preserved at 4°C were damaged *via* microtubule fragility	Surviving cells proliferated and secreted key proteins normally
Khan et al. (32)	Human fetal RPE	CCS	7 days	MEM-based	4, 16, 20, 24, 28, and 37°C	The three lowest storage temperatures generally showed fewer dead cells compared to the three highest storage temperatures.	4 and 16°C storage resulted in best morphology. Membrane blebbing, intercellular distance, and loss of intercellular contact was seen at higher storage temperatures.	No storage temperature expressed differentiation markers in a consistently favorable fashion.
Pasovic et al. (33)	ARPE-19	CCS	7 days	MEM-based	4, 16, and 37°C	Gene expression analysis showed that 16°C storage resulted in highest expression of cell survival genes.	NA	Gene expression analysis suggested that 37°C resulted in cell cycle arrest. This was not observed in the 4 and 16°C groups.
Pasovic et al. (34)	ARPE-19	CCS	7 days	MEM-based	4, 16, and 37°C	NA	NA	Expression of genes related to pigmentation, ion transport, and visual cycle was almost similar among the various storage groups.
Pasovic et al. (35)	ARPE-19	CCS	7 days	MEM-based	4, 8, 12, 16, 20, 24, 28, 32, and 37°C	16 and 20°C were superior for cell survival.	Optimal storage temperatures for morphology: 12, 16, and 20°C	12, 16, and 20°C were superior in maintaining differentiated phenotype compared to other temperatures.
**Conjunctival epithelial cells**								
Eidet et al. (36)	Conjunctival epithelial cells	CCS	4–7 days	MEM-based	4, 8, 12, 16, 20, 24, 28, 32, and 37 °C	Viability was best preserved at 12°C storage.	For longer storage periods, i.e., 7 days, storage temperatures below 12°C appeared more suitable.	Storage temperatures above 12°C showed higher metabolic consumption compared to lower storage temperatures.
Vasania et al. (37)	Conjunctival epithelial cells	Cell suspensions	2–4 days	DMEM/F12-based	2–8°C	Viability decreased gradually as storage time increased—from 92% viability following 6 h storage to 82% viability after 48 h storage.	Morphology was maintained throughout the storage period.	Specific phenotypic markers were not studied; however, cell attachment was described as “good” in all storage durations.
Eidet et al. (38)	Conjunctival epithelial cells	CCS on amniotic membrane	4–7 days	HEPES-MEM and Optisol-GS	23°C	Viability was well-preserved in both storage media.	Ultrastructure integrity was well-preserved during 4-day storage. Epithelial detachment was observed following 7-day storage.	Expression of key phenotypic markers remained unchanged in both storage media.
**Corneal/limbal epithelial cells**								
Jackson et al. (39)	Limbal epithelial cells	CCS	4 days	Optisol-GS	4 vs. 23°C	23°C storage was better in maintaining cell viability compared to storage at 4°C.	Detachment of basal cells from the underlying membrane was observed in cells stored at 4°C.	In contrast to 4°C storage, expression of stem cells and proliferation markers was maintained at pre-storage levels during storage at 23°C.
Utheim et al., (40)	Limbal epithelial cells	CCS	Up to 7 days	Optisol-GS	4°C	Genes associated with cell death and necrosis were upregulated following 4 and 7 days of storage.	NA	Gene expression deviated from the control group as storage duration increased.
Utheim et al., (41)	Limbal explants	CCS	Up to 7 days	Quantum 286 medium or MEM-based medium	23°C	Cell viability was preserved during storage in both media.	No substantial loss of cell layer thickness was observed during storage.	Albeit somewhat reduced, the immature phenotype of cells was preserved during storage.
Utheim et al., (42)	Limbal epithelial cells cultured on amniotic membrane	CCS	Up to 21 days	DMEM-based medium	23°C	Viability was 88% after 14 days storage and 53% after 21 days of storage compared to unstored control.	Multilayered tissue anatomy was preserved in 70% of cultures following 14 days of storage but lost after 21 days of storage.	A less differentiated phenotype was maintained throughout the storage period.
Raeder et al. (43)	Limbal epithelial cells cultured on amniotic membrane	CCS	7 days	Optisol-GS or DMEM-based medium	Optisol-GS 5°C. DMEM-based medium 23 and 31°C.	Although the differences were statistically insignificant, the number of apoptotic cells appeared to correlate with higher storage temperature.	Storage in a DMEM-based medium at 23°C was superior in preserving the original layered structure of the stored cells.	Cells remained undifferentiated in all storage conditions.
**Oral keratinocytes**								
Islam et al. (44)	Oral keratinocytes	CCS	7 days	MEM-based	4, 8, 12, 16, 20, 24, 28, 32, and 37°C	Storage at 12°C maintained the highest number of live cells.	The midrange temperature groups of 12, 16, and 20°C resulted in the best morphology.	Storage temperatures between 4 and 24°C resulted in better preservation of phenotypical markers. Cell metabolism was proportional to storage temperature.
Oie et al. (7)	Oral mucosal epithelial cells	CCS	12 h	HBSS-based	35°C	Viability, morphology, and key phenotypical markers were adequately preserved during transportation.		
Lee et al. (45)	Oral keratinocytes	CCS	Up to 72 h	Keratinocyte growth medium, saline, and DMEM with or without albumin supplementation	4°C or room temperature	Highest cell viability was obtained in saline or DMEM both supplemented with 10% serum albumin at 4°C or at room temperature.	NA	NA
**Seminiferous epithelial cells**								
Faes and Goossens (46)	Seminiferous epithelium	Biopsy specimens	3 days	DMEM/F12	4°C, room temperature, and 37°C	No significant difference was observed in cell viability; however, storage for 3 days at 37°C showed a higher number of apoptotic cells compared to control.	No significant morphological changes were observed.	Storage temperature did not appear to affect the number of spermatogonia in samples.

*The table is sectioned based on cell type, and the rows are sorted by year of publication (descending). CCS, cultured cell sheets; MEM, minimum essential medium; CES, cultured epidermal cell sheets; CnT Prime, a commercially available, fully defined, animal component-free culture medium; DMEM, Dulbecco's modified Eagle medium; DMEM/F12, 1:1 mixture of DMEM and Ham's F-12 medium; ARPE-19, adult human retinal pigment epithelial cell line 19; hiPSC-RPE, human induced pluripotent stem cell-derived retinal pigment epithelium; RPE, retinal pigment epithelium; HEPES, 4-(2-hydroxyethyl)-1-piperazineethanesulfonic acid; Optisol-GS, a commercially available corneal storage medium containing gentamicin and streptomycin; HBSS, Hanks' Balanced Salt Solution*.

## Results

Results are presented in Table 1.

## Discussion

**Figure 3:**
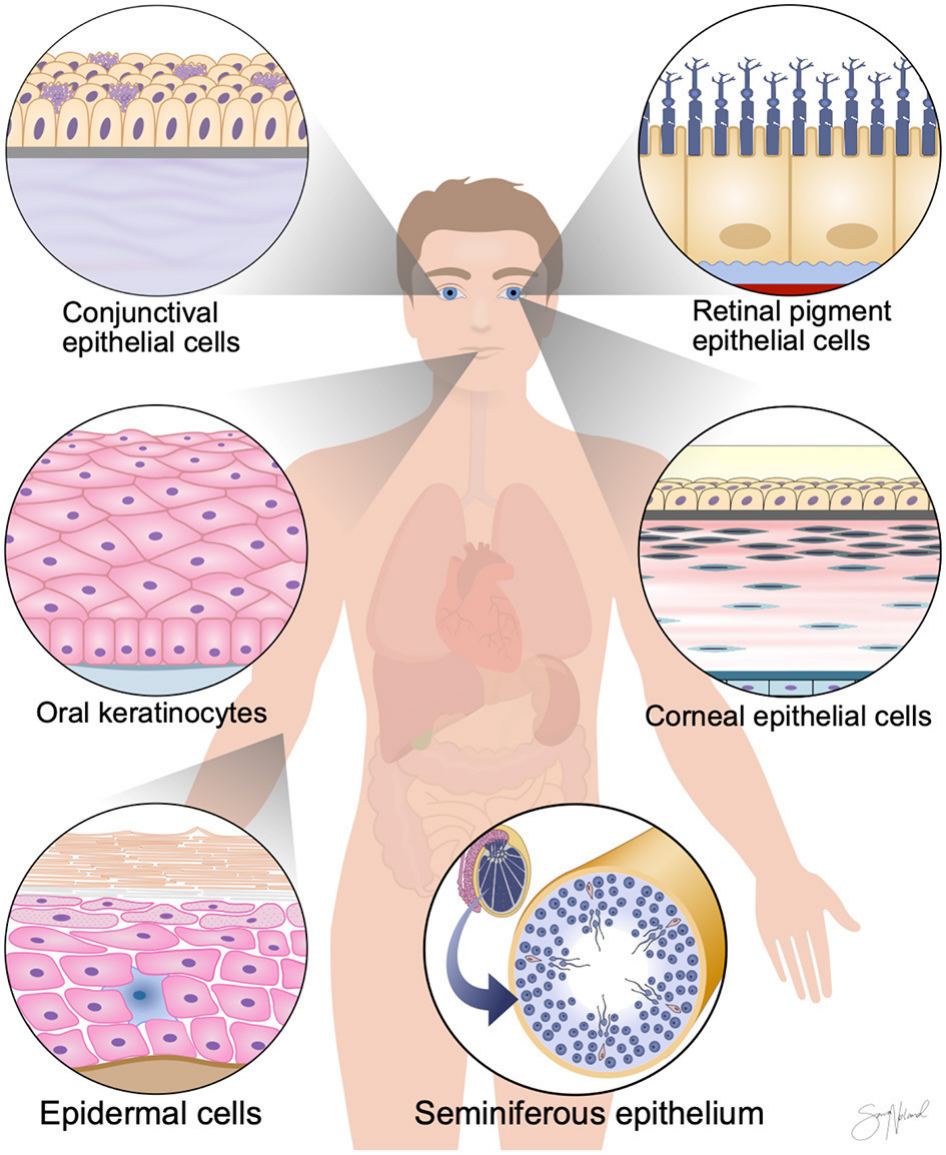
Cell-based regenerative medicine therapies require the development of simple and cost-effective non-freezing preservation methods. Here, we review publications from the last two decades investigating above-freezing point storage of human epithelial cells. The literature search uncovered publications on epidermal cells, retinal pigment epithelial cells, conjunctival epithelial cells, corneal/limbal epithelial cells, oral keratinocytes, and seminiferous epithelial cells.

Epithelium “covers or lines body surfaces and forms the functional units of secretory glands” (47). Epithelial cells are classified based on their anatomical location, the shape of the individual cell (squamous, cuboidal, or columnar), and the arrangement of cells in one or more layers (simple epithelia or stratified epithelia) (47). In this review, we summarize work on storage temperatures for above-freezing point storage of human epithelial cells for regenerative medicine purposes. The literature search uncovered publications on epidermal cells, retinal pigment epithelial (RPE) cells, conjunctival epithelial cells, corneal/limbal epithelial cells, oral keratinocytes, and seminiferous epithelial cells (Figure 3).

### Epidermal Cells

Refrigeration remains the preferred method for the short-term storage of skin grafts. A survey performed among plastic surgery centers across Europe confirmed that split-thickness skin grafts are routinely stored for up to 10 days at 4°C in saline-moisturized gauze (26). Histologic evaluations have shown no major macroscopic or microscopic alterations within skin grafts stored for 7 days at 4°C (26). Seet et al. (28) successfully stored a tissue-engineered skin construct composed of keratinocytes and fibroblasts at 4°C for 3 days without observing a major reduction in cell viability. However, cell viability appears to decline with increased storage duration (26). Li et al. (27) also reported that skin cell viability declines with prolonged storage. Despite testing four different storage media, they showed that viability was reduced to 50% by storage day 14. By storage day 28, viability was <5% across all storage groups (27). Closely related to viability of the skin graft is the colony-forming efficiency of keratinocytes, which was inversely correlated with storage time (27). Hence, the prevailing evidence on skin graft storage at 4°C indicates a storage time of about 7 days, with decreasing viability with increasing storage time.

In our literature search, the earliest report deviating from skin storage at 4°C was a publication by Robb et al. (29) in 2001. In this study, the authors reported better preservation of tissue anatomy when skin grafts were stored at room temperature compared to 4°C for up to 21 days. Importantly, they replaced the storage media every 3 days and thus provided the cells continuous nutrition, which is impractical when transporting grafts. Therefore, we consider this study closer to organ culture rather than cell storage.

When discussing skin storage, a distinction between skin grafts and cultured cells appears to be reasonable, as these two tissues differ considerably in origin, handling, and tissue anatomy.

Whereas clinical experience and scientific evidence agree on 4°C storage as the preferred storage temperature for short-term storage of skin grafts, this is not the case when considering cultured cells. Jackson et al. (23–25) have published three reports on the short-term hypothermic storage of cultured epidermal cell sheets (CES). In the first study, they stored cells for 14 days and reported a tendency of better viability in cells stored at higher temperatures (24, 28, 32, and 37°C) compared to cells stored at lower temperatures (4, 8, 12, 16, and 20°C) (25). However, cell death was most prominent at the extremes of the storage temperatures studied, i.e., 4 and 37°C. In preserving morphology, storage at 12 and 16°C appeared superior compared to other temperatures. In their second study, cells were stored for the same time period and at the same storage temperatures, but differentiation was studied more thoroughly (24). The authors concluded that the undifferentiated phenotype, which is desirable in the case of transplantation, was best maintained at the lower end of the abovementioned temperature spectrum, particularly 12°C. In the third study, cells were stored for 7 days at five different storage temperatures (4, 8, 12, 16, and 24°C) (23). This study concluded, based on morphological, phenotypical, cytokine, viability, and reactive oxygen species assays, that storage at 12°C uniquely provided optimal morphology and undifferentiated phenotype. Interestingly, storage at 12°C yielded the highest post-storage viability in the 1-week study compared to the 2-week storage experiments, suggesting a possible 1-week “shelf life” of cultured epidermal cells stored at 12°C. Reppe et al. (22) achieved a post-storage viability higher than unstored control when storing CES in minimum essential medium (MEM) at 12°C for a week. The increase in viability was attributed to cell proliferation during storage, which may suggest that the storage medium formula is of importance. Similarly, Ringstad et al. (21) stored CES at 12°C but for up to 15 days. They reported superior viability when the cells were stored at a pre-confluent stage (i.e., storage was initiated when cell cultures covered 80% of the culture dish). Collectively, data from these reports are in favor of 12°C as the optimal storage temperature for short-term storage of cultured epidermal cells.

Hence, based on the reported literature, we advise a storage temperature of 4°C for skin grafts and 12°C for cultured epidermal cells.

### Retinal Pigment Epithelial Cells

Transplantation of RPE is emerging as a promising treatment alternative for sight-threatening eye diseases such as age-related macular degeneration, Stargardt macular dystrophy, and some forms of retinitis pigmentosa (48). Results from three important clinical trials have been reported in recent years (49–51).

Storage temperature for short-term preservation of RPE cells has been investigated by six studies. One report showed favorable results with storage at 4°C (32), while three studies concluded that 16°C is the most suitable above-0°C storage temperature (30, 31, 35). In addition, two studies suggested that both 4 and 16°C were suitable storage temperatures (33, 34).

In support of 4°C, Khan et al. (32) stored cultured human fetal RPE sheets for 7 days at six different storage temperatures (4, 16, 20, 24, 28, and 37°C). After 7 days of storage, cell viability, morphology, pH, and phenotypic expression of differentiation markers were assessed. No single storage temperature consistently outperformed other storage temperatures across all investigated parameters. However, storage at 4°C best preserved tissue morphology (in comparison to non-stored control cells). A tendency of higher cell death in the three highest storage temperatures (24, 28, and 37°C) was noted.

In support of 16°C, Kitahata et al. (31) investigated the effect of storage temperature on storage of human induced pluripotent stem cell-derived retinal pigment epithelial (hiPSC-RPE) cell suspensions. They tested four different storage temperatures, 4, 16, 25, and 37°C, and demonstrated best viability when using a 16°C storage temperature. They showed that storage at 4°C resulted in microtubule fragility, while 37°C caused cell death due to hypoxia secondary to elevated cell metabolism. Likewise, a study on cultured ARPE-19 cell sheets stored at 4, 8, 12, 16, 20, 24, 28, 32, and 37°C in a MEM-based medium for 7 days also concluded 16°C to be the optimal storage temperature (35). Importantly, this study was carried out on cultured cell sheets and an immortalized RPE cell line, in contrast to Kitahata et al. (31), who stored cell suspensions and hiPSC-RPE, respectively. In a microarray analysis, Pasovic et al. (34) compared gene expression profiles of ARPE-19 cells stored for 1 week in a MEM-based medium at 4, 16, and 37°C. Storage at 4 and 16°C resulted in gene expression most similar to non-stored control, while storage at 37°C significantly altered gene expression. Furthermore, in a later study, they again showed that gene expression following storage at 4°C was closest to control cultures that were not stored (33). Cultures stored at 16 and 37°C displayed much greater change in gene expression. At 37°C, activation of vascular endothelial growth factor (VEGF) was discovered, which is considered disadvantageous in an RPE graft.

Testing a wide range of temperatures has shown that increments in temperature can alter storage outcome. Although both 4 and 16°C storage show favorable results, only one study investigated a storage temperature between these two temperatures. Hence, the true optimal hypothermic storage temperature may hide in this uninvestigated interval. Finally, a direct comparison between the reported studies is not reasonable due to differences in cell types, storage media, and storage duration.

### Conjunctival Epithelial Cells

Transplantation of cultured conjunctival epithelial cells has been reported to improve vision in patients with limbal stem cell deficiency (LSCD) (52), a disorder characterized by deficient or dysfunctional stem cells in the limbal region (53). Three papers describe storage of conjunctival epithelial cells. The first study stored conjunctival epithelial cells cultured on amniotic membrane in MEM containing 4-(2-hydroxyethyl)-1-piperazineethanesulfonic acid (HEPES-MEM) and Optisol-GS at 23°C for 4 and 7 days (38). In this paper, viability and phenotype were maintained for at least 4 days of storage at 23°C (in both media). The second study was a prospective, open-label, single–arm, multicentric clinical trial in which 25 patients underwent autologous conjunctival epithelial cell transplantation (37). The clinical outcome was reported to be satisfactory and without serious adverse effects. The cell grafts were stored and transported for a 48-h period at a temperature interval of 2–8°C before surgery. Investigation of parallel cultures showed that cell attachment and morphology were acceptable throughout the storage period. Cell viability was adversely affected as storage time increased, dropping to 95, 90, 88, and 82% after storage at 6, 12, 24, and 48 h, respectively. The third study evaluated the effects of storage temperature on morphology, viability, cell number, and metabolism of cultured human conjunctival epithelial cells (36). Cells were stored for 4 and 7 days. The following temperatures were investigated: 4, 8, 12, 16, 20, 24, 28, 32, and 37 °C. Here, 12°C storage appeared optimal, as this was the only storage temperature at which viability was preserved following a 7-day storage period. Moreover, total cell number had decreased in all groups, except 12°C. Furthermore, cell morphology was also maintained at this temperature. The authors suggested temperature-related effects on cell metabolism as the primary reason for their findings. Among the three cited studies, only the latter compared different storage temperatures, making it difficult to conclude on an optimal storage temperature.

### Corneal/Limbal Epithelial Cells

A number of reports have been published on the storage of limbal epithelial cells (39, 41–43). Although most are in favor of 23°C storage in an Optisol-GS or a DMEM-based medium, it must be noted that the mentioned studies have only tested 4, 23, and 37°C temperatures. As the preceding subsections discussing other cell types suggest, the temperature interval between 4 and 23°C is highly relevant and should be investigated in future studies.

Transplantation of cultured limbal epithelial cells is a therapy for LSCD (54). The ability of cultured limbal epithelial grafts to proliferate and generate a healthy population of cells is therefore critical. None of the reported publications investigated whether storage temperature affects stemness potential or the percentage of holoclones, meroclones, and paraclones that can be isolated from a biopsy and propagated *in vitro*. To conduct a correlation between storage temperature and stemness potential is therefore currently not possible. However, Jackson et al. (39) reported that expression of the stem cell marker ABCG2 was significantly reduced in the 4°C storage group compared to 23°C storage. Utheim et al. (41) also demonstrated that 23°C storage retained an immature phenotype in cultured limbal epithelial cells.

### Oral Keratinocytes

Cultured oral keratinocytes can be used to reconstruct damaged corneas and thus restore vision (55). Hypothermic storage of oral keratinocytes has been reported by Lee et al. (45), Oie et al. (7), and Islam et al. [2015]. Lee et al. (45) stored cell suspensions of primary human oral keratinocytes for 24, 48, and 72 h at 4°C and at room temperature. No significant decline in viability was observed for at least 48 h. Oie et al. (7) described a container that can maintain sterility, temperature stability, and air pressure during cell transportation. Using this custom-made container, they transported cultured oral mucosal epithelial cells by air for a transportation period of 12 h. Cell viability, morphology, phenotype, and sterility parameters were maintained during transportation. This investigation on both storage and transportation of cell products (considering practical challenges) is an excellent example of how future studies could be designed. Islam et al. (44) tested the following temperatures, 4, 8, 12, 16, 20, 24, 28, 32, and 37°C, over a storage period of 7 days. Relative to non-stored control cells, a high percentage of viable cells was retained only in the groups stored at 12 and 16°C. Morphology was preserved at 12, 16, and 20°C storage.

### Other Epithelia

Faes and Goossens (46) studied how temperature affects storage of testicular tissue, including seminiferous epithelium. The tissue samples were stored at 4°C, room temperature, and 37°C. Tissue quality (judged by histology, immunohistochemistry, and apoptosis) was maintained at all temperatures following a 3-day storage period. However, in contrast to 4°C and room temperature, they found that 37°C storage caused a significant increase in apoptotic cells.

## Conclusion and Future Perspectives

In this review, we summarized the work investigating storage temperature for above-freezing point storage of human epithelial cells such as epidermal cells, RPE, conjunctival epithelial cells, corneal/limbal epithelial cells, oral keratinocytes, and seminiferous epithelial cells (Table 2). Such a summary is challenging for several reasons. First, the epithelial cells examined not only reside in different anatomical locations, they also differ in state (cultured cells vs. grafts; cell lines vs. primary cells; cell sheets vs. cell suspensions). Second, each study employed its own distinct set of cell culture media and storage media. Third, parameters of interest varied from study to study, e.g., while some focused on viability, others emphasized phenotypic and functional characterization. In sum, these variations do not allow for a fair comparison between the studies.

**Table 2:** The table summarizes findings of the literature review of storage temperature for above-freezing point short-term storage of human epithelial cells.

**Temperature recommendations for the short-term storage of**	
**various epithelial cells**	
Skin grafts	4°C
Cultured epidermal cells	12°C
Retinal pigment epithelial cells	Between 4 and 16°C
Conjunctival epithelial cells	Insufficient evidence
Corneal/limbal epithelial cells	23°C
Oral keratinocytes	Insufficient evidence
Seminiferous epithelial cells	Both room temperature and 4°C

Nevertheless, some general tendencies can be observed. For instance, several studies across different epithelial cell types incline toward temperatures between 4 and 16°C being suitable as short-term storage temperatures. Correspondingly, almost all studies that investigated 37°C concluded that this storage temperature was suboptimal. Another common observation (when analyzing studies investigating storage duration) is that storage time typically should not exceed 7–10 days, as viability tends to decrease dramatically after this duration. Finally, the importance of the type of storage medium and its composition was highlighted by some of the studies. The topic of storage media deserves a separate review.

Future efforts should be directed toward investigation of clinical outcomes after transplantation of stored cell products.

## Data Availability Statement

The original contributions presented in the study are included in the article/Supplementary Material, further inquiries can be directed to the corresponding author/s.

## Author Contributions

AK performed the literature review and drafted the manuscript. TU, CJ, KT, and JE edited the manuscript. All authors contributed to the article and approved the submitted version.

## Conflict of Interest

JE and TU hold a patent on the use of sericin in culture media (European Patent Number EP3317404), filed by Inven2 (the technology transfer office of the University of Oslo and Oslo University Hospital). The authors declare that the research was conducted in the absence of any commercial or financial relationships that could be construed as a potential conflict of interest. The reviewer DT declared a past co-authorship with several of the authors to the handling editor.

## Publisher's Note

All claims expressed in this article are solely those of the authors and do not necessarily represent those of their affiliated organizations, or those of the publisher, the editors and the reviewers. Any product that may be evaluated in this article, or claim that may be made by its manufacturer, is not guaranteed or endorsed by the publisher.

## References

[1] Yang R Liu F Wang J Chen X Xie J Xiong K. Epidermal stem cells in wound healing and their clinical applications. Stem Cell Res Ther. (2019) 10:229. 10.1186/s13287-019-1312-z 31358069 PMC6664527

[2] Saghizadeh M Kramerov AA Svendsen CN Ljubimov AV. Concise review: stem cells for corneal wound healing. Stem Cells. (2017) 35:2105–114. 10.1002/stem.2667 28748596 PMC5637932

[3] Rogovaya OS Fayzulin AK Vasiliev AV Kononov AV Terskikh VV. Reconstruction of rabbit urethral epithelium with skin keratinocytes. Acta Nat. (2015) 7:70–7. 10.32607/20758251-2015-7-1-70-77 25927003 PMC4410397

[4] Zarbin M Sugino I Townes-Anderson E. Concise review: update on retinal pigment epithelium transplantation for age-related macular degeneration. Stem Cells Transl Med. (2019) 8:466–77. 10.1002/sctm.18-0282 30748126 PMC6477002

[5] Van Buskirk RG Baust JM Snyder KK Mathew AJ Baust JG. Hypothermic storage and cryopreservation. BioProcess Int. (2004) 2. Available online at: http://www.bioprocessintl.com/wp-content/uploads/bpi-content/0210ar06_76760a.pdf

[6] Michel SG Lamuraglia Ii GM Madariaga MLL Anderson LM. Innovative cold storage of donor organs using the Paragonix Sherpa Pak ™ devices. Heart Lung Vessels. (2015) 7:246−55. 26495271 PMC4593023

[7] Oie Y Nozaki T Takayanagi H Hara S Hayashi R Takeda S . Development of a cell sheet transportation technique for regenerative medicine. Tissue Eng Part C Methods. (2014) 20:373–82. 10.1089/ten.tec.2013.0266 24044382 PMC4005488

[8] Colvert A Coty H. Cryopreservation: Technologies, Applications and Risks/Outcomes. Hauppauge: Nova Science Publishers, Incorporated (2013).

[9] Hunt CJ. Cryopreservation of human stem cells for clinical application: a review. Transfus Med Hemother. (2011) 38:107–23. 10.1159/000326623 21566712 PMC3088734

[10] Xu X Cowley S Flaim CJ James W Seymour L Cui Z. The roles of apoptotic pathways in the low recovery rate after cryopreservation of dissociated human embryonic stem cells. Biotechnol Prog. (2010) 26:827–37. 10.1002/btpr.368 20077485 PMC3596802

[11] Chakraborty N Biswas D Parker W Moyer P Elliott GD. A role for microwave processing in the dry preservation of mammalian cells. Biotechnol Bioeng. (2008) 100:782–96. 10.1002/bit.21801 18318445

[12] Chakraborty N Chang A Elmoazzen H Menze MA Hand SC Toner M. A spin-drying technique for lyopreservation of mammalian cells. Ann Biomed Eng. (2011) 39:1582–91. 10.1007/s10439-011-0253-1 21293974

[13] Puhlev I Guo N Brown DR Levine F. Desiccation tolerance in human cells. Cryobiology. (2001) 42:207–17. 10.1006/cryo.2001.2324 11578120

[14] Bakaltcheva I Leslie S Macdonald V Spargo B Rudolph A. Reversible cross-linking and CO treatment as an approach in red cell stabilization. Cryobiology. (2000) 40:343–59. 10.1006/cryo.2000.2257 10924266

[15] Fowler A Toner M. Cryo-injury and biopreservation. Ann N Y Acad Sci. (2005) 1066:119–35. 10.1196/annals.1363.010 16533923

[16] Chen B Wright B Sahoo R Connon CJ. A novel alternative to cryopreservation for the short-term storage of stem cells for use in cell therapy using alginate encapsulation. Tissue Eng Part C Methods. (2013) 19:568–76. 10.1089/ten.tec.2012.0489 23199013

[17] Wright B Cave RA Cook JP Khutoryanskiy VV Mi S Chen B . Enhanced viability of corneal epithelial cells for efficient transport/storage using a structurally modified calcium alginate hydrogel. Regen Med. (2012) 7:295–307. 10.2217/rme.12.7 22594324

[18] Wright B Mi S Connon CJ. Towards the use of hydrogels in the treatment of limbal stem cell deficiency. Drug Discov Today. (2013) 18:79–86. 10.1016/j.drudis.2012.07.012 22846850 PMC4046643

[19] Wojcik G Ferrari S Romano V Ponzin D Ahmad S Parekh M. Corneal storage methods: considerations and impact on surgical outcomes. Exp Rev Ophthalmol. (2020) 16:1–9. 10.1080/17469899.2021.1829476

[20] Armitage WJ. Preservation of human cornea. Transfus Med Hemother. (2011) 38:143–7. 10.1159/000326632 21566714 PMC3088736

[21] Ringstad H Reppe S Schøyen TH Tønseth KA Utheim TP Jackson CJ. Stem cell function is conserved during short-term storage of cultured epidermal cell sheets at 12°C. PLoS ONE. (2020) 15:e0232270. 10.1371/journal.pone.0232270 32433698 PMC7239464

[22] Reppe S Jackson CJ Ringstad H Tønseth KA Bakke H Eidet JR . High throughput screening of additives using factorial design to promote survival of stored cultured epithelial sheets. Stem Cells Int. (2018) 2018:1–9. 10.1155/2018/6545876 30581473 PMC6276401

[23] Jackson CJ Reppe S Eidet JR Eide L Tønseth KA Bergersen LH . Optimization of storage temperature for retention of undifferentiated cell character of cultured human epidermal cell sheets. Sci Rep. (2017) 7:8206. 10.1038/s41598-017-08586-7 28811665 PMC5557837

[24] Jackson C Eidet JR Reppe S Aass HC Tønseth KA Roald B . Effect of storage temperature on the phenotype of cultured epidermal cells stored in xenobiotic-free medium. Curr Eye Res. (2016) 41:757–68. 10.3109/02713683.2015.1062113 26398483

[25] Jackson C Aabel P Eidet JR Messelt EB Lyberg T Von Unge M . Effect of storage temperature on cultured epidermal cell sheets stored in xenobiotic-free medium. PLoS ONE. (2014) 9:e105808. 10.1371/journal.pone.0105808 25170754 PMC4149437

[26] Knapik A Kornmann K Kerl K Calcagni M Contaldo C Vollmar B . Practice of split-thickness skin graft storage and histological assessment of tissue quality. J Plast Reconstruct Aesthet Surg. (2013) 66:827–34. 10.1016/j.bjps.2013.02.003 23545226

[27] Li Z Overend C Maitz P Kennedy P. Quality evaluation of meshed split-thickness skin grafts stored at 4°C in isotonic solutions and nutrient media by cell cultures. Burns. (2012) 38:899–907. 10.1016/j.burns.2012.02.002 22385642

[28] Seet WT Maarof M Khairul Anuar K Chua K.-H Ahmad Irfan AW . Shelf-life evaluation of bilayered human skin equivalent, MyDerm™. PLoS ONE. (2012) 7:e40978. 10.1371/annotation/44cd1027-1f9e-4843-b013-84ca45ae942f 22927903 PMC3426510

[29] Robb EC Bechmann N Plessinger RT Boyce ST Warden GD Kagan RJ. Storage media and temperature maintain normal anatomy of cadaveric human skin for transplantation to full-thickness skin wounds. J Burn Care Rehabil. (2001) 22:393–6. 10.1097/00004630-200111000-00008 11761390

[30] Islam R Corraya RM Pasovic L Khan AZ Aass HCD Eidet JR . The effects of prolonged storage on ARPE-19 cells stored at three different storage temperatures. Molecules. (2020) 25:5809. 10.3390/molecules25245809 33317020 PMC7763992

[31] Kitahata S Tanaka Y Hori K Kime C Sugita S Ueda H . Critical functionality effects from storage temperature on human induced pluripotent stem cell-derived retinal pigment epithelium cell suspensions. Sci Rep. (2019) 9:2891. 10.1038/s41598-018-38065-6 30814559 PMC6393435

[32] Khan AZ Utheim TP Reppe S Sandvik L Lyberg T Roald BB . Cultured human retinal pigment epithelial (hRPE) sheets: a search for suitable storage conditions. Microsc Microanal. (2018) 24:147–55. 10.1017/S1431927618000144 29637873

[33] Pasovic L Eidet JR Olstad OK Chen DF Lyberg T Utheim TP. Impact of storage temperature on the expression of cell survival genes in cultured ARPE-19 cells. Curr Eye Res. (2017) 42:134–44. 10.3109/02713683.2016.1145236 27259952

[34] Pasovic L Eidet JR Brusletto BS Lyberg T Utheim TP. Effect of storage temperature on key functions of cultured retinal pigment epithelial cells. J Ophthalmol. (2015) 2015:263756. 10.1155/2015/263756 26448872 PMC4584032

[35] Pasovic L Utheim TP Maria R Lyberg T Messelt EB Aabel P . Optimization of storage temperature for cultured ARPE-19 cells. J Ophthalmol. (2013) 2013:216359. 10.1155/2013/216359 24251032 PMC3819763

[36] Eidet JR Utheim ØA Islam R Lyberg T Messelt EB Dartt DA . The impact of storage temperature on the morphology, viability, cell number and metabolism of cultured human conjunctival epithelium. Curr Eye Res. (2015) 40:30–9. 10.3109/02713683.2014.909497 24750037 PMC4206676

[37] Vasania VS Hari A Tandon R Shah S Haldipurkar S Shah S . Transplantation of autologous *ex vivo* expanded human conjunctival epithelial cells for treatment of pterygia: a prospective open-label single arm multicentric clinical trial. J Ophthalmic Vis Res. (2014) 9:407–16. 10.4103/2008-322X.150800 25709763 PMC4329698

[38] Eidet JR Utheim OA Raeder S Dartt DA Lyberg T Carreras E . Effects of serum-free storage on morphology, phenotype, and viability of *ex vivo* cultured human conjunctival epithelium. Exp Eye Res. (2012) 94:109–16. 10.1016/j.exer.2011.11.015 22154551

[39] Jackson CJ Pasovic L Raeder S Sehic A Roald B De La Paz MF . Optisol-GS storage of cultured human limbal epithelial cells at ambient temperature is superior to hypothermic storage. Curr Eye Res. (2020) 12:1–7. 10.1080/02713683.2020.1770295 32578462

[40] Utheim TP Salvanos P Utheim ØA. Transcriptome analysis of cultured limbal epithelial cells on an intact amniotic membrane following hypothermic storage in optisol-GS. J Funct. (2016). 7:4. 10.3390/jfb7010004 26901233 PMC4810063

[41] Utheim O Islam R Lyberg T Roald B Eidet JR De La Paz MF . Serum-free and xenobiotic-free preservation of cultured human limbal epithelial cells. PLoS ONE. (2015) 10:e0118517. 10.1371/journal.pone.0118517 25734654 PMC4348416

[42] Utheim TP Raeder S Utheim ØA de la Paz M Roald B Lyberg T. Sterility control and long-term eye-bank storage of cultured human limbal epithelial cells for transplantation. Br J Ophthalmol. (2009) 93:980–3. 10.1136/bjo.2008.149591 19211610

[43] Raeder S Utheim TP Utheim OA Nicolaissen B Roald B Cai Y . Effects of organ culture and Optisol-GS storage on structural integrity, phenotypes, and apoptosis in cultured corneal epithelium. Invest Ophthalmol Vis Sci. (2007) 48:5484–93. 10.1167/iovs.07-0494 18055796

[44] Islam R Jackson C Eidet JR Messelt EB Corraya RM Lyberg T . Effect of storage temperature on structure and function of cultured human oral keratinocytes. PLoS ONE. (2015) 10:e0128306. 10.1371/journal.pone.0128306 26052937 PMC4459984

[45] Lee EJ Lee SA Kim J. The effect of human serum albumin on the extended storage of human oral keratinocyte viability under mild hypothermia. Cryobiology. (2005) 50:103–11. 10.1016/j.cryobiol.2004.12.001 15710374

[46] Faes K Goossens E. Short-term storage of human testicular tissue: effect of storage temperature and tissue size. Reprod Biomed Online. (2017) 35:180–8. 10.1016/j.rbmo.2017.04.011 28583375

[47] Kierszenbaum AL Tres L. Histology and Cell Biology: An Introduction to Pathology. E-journal, Amsterdam: Elsevier Health Sciences (2015).

[48] da Cruz L Chen FK Ahmado A Greenwood J Coffey P. RPE transplantation and its role in retinal disease. Prog Retin Eye Res. (2007) 26:598–635. 10.1016/j.preteyeres.2007.07.001 17920328

[49] Schwartz SD Regillo CD Lam BL Eliott D Rosenfeld PJ Gregori NZ . Human embryonic stem cell-derived retinal pigment epithelium in patients with age-related macular degeneration and Stargardt's macular dystrophy: follow-up of two open-label phase 1/2 studies. Lancet. (2015) 385:509–16. 10.1016/S0140-6736(14)61376-3 25458728

[50] Kashani AH Lebkowski JS Rahhal FM Avery RL Salehi-Had H Dang W . A bioengineered retinal pigment epithelial monolayer for advanced, dry age-related macular degeneration. Sci Transl Med. (2018) 10:1–10. 10.1126/scitranslmed.aao4097 29618560

[51] da Cruz L Fynes K Georgiadis O Kerby J Luo YH Ahmado A . Phase 1 clinical study of an embryonic stem cell-derived retinal pigment epithelium patch in age-related macular degeneration. Nat Biotechnol. (2018) 36:328–37. 10.1038/nbt.4114 29553577

[52] Ricardo JR Cristovam PC Filho PA Farias CC De Araujo AL Loureiro RR . Transplantation of conjunctival epithelial cells cultivated *ex vivo* in patients with total limbal stem cell deficiency. Cornea. (2013) 32:221–8. 10.1097/ICO.0b013e31825034be 22580434

[53] Le Q Xu J Deng SX. The diagnosis of limbal stem cell deficiency. Ocul Surf. (2018) 16:58–69. 10.1016/j.jtos.2017.11.002 29113917 PMC5844504

[54] Pellegrini G Traverso CE Franzi AT Zingirian M Cancedda R De Luca M. Long-term restoration of damaged corneal surfaces with autologous cultivated corneal epithelium. Lancet. (1997) 349:990–3. 10.1016/S0140-6736(96)11188-0 9100626

[55] Nishida K Yamato M Hayashida Y Watanabe K Yamamoto K Adachi E . Corneal reconstruction with tissue-engineered cell sheets composed of autologous oral mucosal epithelium. N Engl J Med. (2004) 351:1187–96. 10.1056/NEJMoa040455 15371576

