# Corrigendum: Concise Review: Considering Optimal Temperature for Short-Term Storage of Epithelial Cells

**DOI:** 10.3389/fmed.2021.775351

**Published:** 2021-10-05

**Authors:** Ayyad Zartasht Khan, Tor Paaske Utheim, Catherine Joan Jackson, Kim Alexander Tønseth, Jon Roger Eidet

**Affiliations:** ^1^Department of Medical Biochemistry, Oslo University Hospital, Oslo, Norway; ^2^Department of Surgery, Sørlandet Hospital Arendal, Arendal, Norway; ^3^Department of Ophthalmology, Sørlandet Hospital Arendal, Arendal, Norway; ^4^Institute of Clinical Medicine, Faculty of Medicine, University of Oslo, Oslo, Norway; ^5^Department of Oral Biology, Faculty of Dentistry, University of Oslo, Oslo, Norway; ^6^Department of Ophthalmology, Stavanger University Hospital, Stavanger, Norway; ^7^Department of Plastic and Reconstructive Surgery, Oslo University Hospital, Oslo, Norway; ^8^Department of Ophthalmology, Oslo University Hospital, Oslo, Norway; ^9^Ifocus Eye Clinic, Haugesund, Norway

**Keywords:** cell banking, regenerative medicine, storage temperature, cell therapy, storage technologies, transplantation

In the published article, there was an error regarding the affiliations for Ayyad Zartasht Khan. As well as having affiliations “Department of Medical Biochemistry, Oslo University Hospital, Oslo, Norway, Department of Surgery, Sørlandet Hospital Arendal, Arendal, Norway, Department of Ophthalmology, Sørlandet Hospital Arendal, Arendal, Norway,” he should also have the following affiliation: “Institute of Clinical Medicine, Faculty of Medicine, University of Oslo, Oslo, Norway.”

The authors apologize for this error and state that this does not change the scientific conclusions of the article in any way. The original article has been updated.

## Publisher's Note

All claims expressed in this article are solely those of the authors and do not necessarily represent those of their affiliated organizations, or those of the publisher, the editors and the reviewers. Any product that may be evaluated in this article, or claim that may be made by its manufacturer, is not guaranteed or endorsed by the publisher.

