# Supplementary material for: Concise Review: Considering Optimal Temperature for Short-Term Storage of Epithelial Cells
Source: Front Med (Lausanne). 2021 Aug 18;8:686774. doi: 10.3389/fmed.2021.686774 (PMC8416270; doi:10.3389/fmed.2021.686774)
Supplement: Supplementary file 1 [file Data_Sheet_1.docx]

# Appendix

Documentation of the literature search. Embase, Ovid MEDLINE, Cochrane Library, and Google Scholar were searched using the search terms and algorithms listed in each table. ti,ab,kw = terms in either title or abstract or keyword fields.

| Documentation of the Literature Search | |
| --- | --- |
| **Database** | Embase <1974 to 2021 January 18> |
| **Search Date (DD.MM.YYYY)** | 18.01.2021 |
| **Search Terms (number of hits in parenthesis)** | 1 exp epithelium cell/ (531130)  2 (epitheli* adj3 cell*).ti,ab,kw. (275959)  3 epidermis cell/ (8536)  4 ((epidermal or epidermis) adj3 cell*).ti,ab,kw. (24400)  5 keratinocyte/ (53616)  6 (keratinocyte* or ((cornified or malpighian or skin*) adj3 cell*)).ti,ab,kw. (78594)  7 ((limbal or corneal epitheli*) adj3 cell*).ti,ab,kw. (7203)  8 retina pigment cell/ (553)  9 (retina* adj pigment adj3 cell*).ti,ab,kw. (7486)  10 1 or 2 or 3 or 4 or 5 or 6 or 7 or 8 or 9 (704142)  11 exp "preservation and storage"/ (144549)  12 (stor* or preservat*).ti,ab,kw. (557406)  13 11 or 12 (613787)  14 (((short* or intermediate) adj2 term) or temporary or provision* or provisory).ti,ab,kw. (483975)  15 10 and 13 and 14 (273)  16 limit 15 to embase (190) |
| **Number of Hits Retrieved** | 190 (before removal of duplicates) |

| **Database** | Ovid MEDLINE(R) ALL <1946 to January 18, 2021> |
| --- | --- |
| **Search Date (DD.MM.YYYY)** | 18.01.2021 |
| **Search Terms (number of hits in parenthesis)** | 1 exp Epithelial Cells/ (546200)  2 (epitheli* adj3 cell*).ti,ab,kf. (217663)  3 exp Epidermal Cells/ (40862)  4 ((epidermal or epidermis) adj3 cell*).ti,ab,kf. (21433)  5 Keratinocytes/ (25661)  6 (keratinocyte* or ((cornified or malpighian or skin*) adj3 cell*)).ti,ab,kf. (57418)  7 ((limbal or corneal epitheli*) adj3 cell*).ti,ab,kf. (5441)  8 (retina* adj pigment adj3 cell*).ti,ab,kf. (5802)  9 1 or 2 or 3 or 4 or 5 or 6 or 7 or 8 (732864)  10 exp Preservation, Biological/ (75005)  11 (stor* or preservat*).ti,ab,kf. (453812)  12 10 or 11 (498395)  13 (((short* or intermediate) adj2 term) or temporary or provision* or provisory).ti,ab,kf. (370875)  14 9 and 12 and 13 (249) |
| **Number of Hits Retrieved** | 249 (before removal of duplicates) |

| **Database** | Cochrane Library |
| --- | --- |
| **Search Date (DD.MM.YYYY)** | 18.01.2021 |
| **Search Terms (number of hits in parenthesis)** | #1 MeSH descriptor: [Epithelial Cells] explode all trees 1964  #2 (epitheli* NEAR/2 cell*) 2041  #3 MeSH descriptor: [Epidermal Cells] explode all trees 190  #4 ((epidermal or epidermis) NEAR/2 cell*) 336  #5 MeSH descriptor: [Keratinocytes] this term only 137  #6 (keratinocyte* or ((cornified or malpighian or skin*) NEAR/2 cell*)) 1325  #7 ((limbal or corneal epitheli*) NEAR/2 cell*) 2695  #8 (retina* NEXT pigment NEAR/2 cell*) 20  #9 #1 or #2 or #3 or #4 or #5 or #6 or #7 or #8 5662  #10 MeSH descriptor: [Preservation, Biological] explode all trees 948  #11 ((stor* or preservat*)):ti,ab,kw 19795  #12 #10 or #11 20214  #13 (((short* or intermediate) NEAR/1 term) or temporary or provision* or provisory) 55575  #14 #9 and #12 and #13 20 |
| **Number of Hits Retrieved** | 20 (before removal of duplicates) |

| **Database** | Google Scholar |
| --- | --- |
| **Search Date (DD.MM.YYYY)** | 18.01.2021 |
| **Search Terms (number of hits in parenthesis)** | 1: "epithelial cells" "short term storage" 2: "epidermal cells" "short term storage" 3: keratinocytes "short term storage" 4: limbal cells "short term storage" 5: retinal pigment cells "short term storage" |
| **Number of Hits Retrieved** | 250 (before removal of duplicates) |
| **Comments** | Because elaborate search terms are less effective in Google Scholar Searches, we performed five different searches as indicated above. For each search, the first 50 hits (with the highest Google Page Ranks) were included. |
